# The Volume and Density of Epicardial Adipose Tissue Measured by Computed Tomography as Markers of the Effects of Cardiovascular Disease Risk Factors

**DOI:** 10.3390/jcdd13060231

**Published:** 2026-05-28

**Authors:** Paweł Gać, Przemysław Cheładze, Rafał Poręba

**Affiliations:** 1 Centre for Diagnostic Imaging, 4th Military Hospital, 50-981 Wroclaw, Poland; 2 Department of Environmental Health, Occupational Medicine and Epidemiology, Wroclaw Medical University, 50-345 Wroclaw, Poland; 3 Department of Biological Principles of Physical Activity, Wroclaw University of Health and Sport Sciences, 51-612 Wroclaw, Poland

**Keywords:** cardiovascular disease risk factors, cardiovascular health, coronary computed tomography angiography, dyslipidemia, epicardial adipose tissue, obesity, overweight, type 2 diabetes

## Abstract

Background/Objectives: Epicardial adipose tissue (EAT), a visceral fat depot located between the myocardium and the visceral pericardium, has emerged as a potential imaging biomarker of cardiometabolic dysfunction and cardiovascular health. This study aimed to evaluate whether computed tomography (CT)-derived epicardial adipose tissue volume (EATV) and epicardial adipose tissue density (EATD) may serve as markers of the effects of major cardiovascular disease risk factors. Methods: This cross-sectional study included 105 participants (mean age: 62.12 ± 11.20 years; 58.1% women) examined with coronary computed tomography angiography. Clinical evaluation included anthropometric assessment, blood pressure measurement, laboratory testing, and questionnaire-based smoking status. EAT was assessed using semi-automatic segmentation on a dedicated post-processing workstation, with visual verification and manual correction, when necessary, within an attenuation range of −190 to −30 HU. Two parameters were analyzed: EATV and EATD. Results: EATV was significantly higher in men, participants with overweight/obesity, participants with dyslipidemia, and participants with type 2 diabetes. EATD was significantly lower in participants with overweight/obesity, dyslipidemia, and type 2 diabetes. Both EATV and EATD were associated with the total number of CVRFs. Correlation analysis showed that EATV was positively associated with age (r = 0.20, *p* = 0.04), BMI (r = 0.54, *p* = 0.01), fasting glycemia (r = 0.46, *p* = 0.01), and total CVRF number (r = 0.51, *p* = 0.01), whereas EATD was negatively associated with age (r = −0.26, *p* = 0.01), BMI (r = −0.40, *p* = 0.01), triglycerides (r = −0.44, *p* = 0.02), fasting glycemia (r = −0.49, *p* = 0.01), and total CVRF number (r = −0.40, *p* = 0.01). In regression analysis, older age, higher BMI, male gender, and dyslipidemia were independent risk factors for higher EATV, while dyslipidemia and type 2 diabetes were independent risk factors for lower EATD. Conclusions: Greater exposure to major cardiovascular disease risk factors was associated with higher EAT volume and lower EAT density, supporting the role of EAT as a structural and metabolic marker of the effects of cardiovascular disease risk factors on cardiovascular health.

## 1. Introduction

Cardiovascular disease (CVD) remains the leading cause of morbidity and mortality worldwide and continues to impose a substantial clinical and public health burden despite advances in prevention, pharmacotherapy, and imaging-based diagnostics [1,2,3]. The risk of CVD is determined by both non-modifiable and modifiable cardiovascular risk factors, with the latter including arterial hypertension, dyslipidemia, smoking, obesity, impaired glucose metabolism, and diabetes mellitus [2,3,4]. These factors do not act in isolation. Rather, their cumulative presence reflects a broader adverse cardiometabolic profile that promotes vascular injury, myocardial remodeling, atherosclerosis, and adverse cardiovascular outcomes.

Although conventional risk assessment models remain indispensable in clinical practice, they are based mainly on demographic, clinical, and biochemical parameters and may only partially reflect the anatomical and metabolic consequences of prolonged exposure to cardiovascular risk factors. For this reason, imaging biomarkers capable of capturing subclinical structural and functional changes are of increasing interest. Among such markers, cardiac adipose tissue, especially epicardial adipose tissue (EAT), has attracted growing attention [5,6,7].

EAT is the visceral adipose tissue located between the myocardium and the visceral pericardium, without a fascial layer separating it from the underlying myocardium and coronary arteries [5,6]. Owing to this intimate anatomical relationship, EAT is not merely a passive fat depot. It is a metabolically active tissue involved in free fatty acid turnover, secretion of adipokines and inflammatory mediators, local immune signaling, and paracrine interactions with the myocardium and coronary vasculature [5,6,7]. Under physiological conditions, EAT may exert protective and energy-buffering functions. However, excessive or metabolically altered EAT has been linked to coronary atherosclerosis, plaque vulnerability, atrial fibrillation, impaired coronary flow, and broader cardiometabolic dysfunction [6,8,9,10,11,12].

Increased EAT may have prognostic significance in patients with coronary artery disease, heart failure, and cardiac arrhythmias, particularly with respect to the occurrence of cardiovascular events [13,14]. In patients with coronary artery disease, increased EAT may predict more advanced atherosclerosis, particularly increased plaque burden, plaque instability, and the presence of hemodynamically significant stenoses in the coronary arteries [15]. In heart failure, EAT has been shown to predict the occurrence of diastolic dysfunction and increased myocardial fibrosis [16]. Among cardiac arrhythmias, the most evidence for the predictive value of EAT has been obtained for atrial fibrillation. Increased EAT around the left atrium may predict atrial remodeling, a higher risk of atrial fibrillation, and recurrence after ablation procedures [17]. The existing literature has demonstrated a predictive association between increased EAT and cardiovascular events, such as myocardial infarction and stroke, as well as hospitalization in cardiology departments and overall and cardiovascular mortality [18]. Beyond its predictive value and use in risk stratification, the amount of EAT may also have other clinical implications. Potentially, the amount of EAT could be a potential therapeutic target [19].

EAT can be assessed using echocardiography, computed tomography (CT), and magnetic resonance imaging (MRI). Echocardiography is widely available and convenient, but it usually measures only EAT thickness and is limited by operator dependence and lower reproducibility. MRI is considered the reference standard for volumetric fat quantification, but its routine use is constrained by cost, accessibility, and examination time. CT, especially when performed as part of coronary computed tomography angiography (CCTA), offers high spatial resolution, good reproducibility, and robust volumetric analysis, making it particularly suitable for EAT assessment in both scientific studies and clinical cohorts [6,20,21].

Importantly, different imaging-derived EAT parameters may reflect different biological aspects of the tissue. EAT thickness is simple to obtain but represents only a local linear measurement. EAT volume (EATV) better captures the overall amount of epicardial fat and is generally considered more informative in CT-based analyses. EAT density (EATD), expressed in Hounsfield units (HU), may reflect tissue composition and metabolic activity, including fibrosis, inflammation, lipid content, and adipocyte remodeling [6,20,21,22,23,24,25]. Thus, volume and density should not be regarded as interchangeable measurements.

Although the association between EAT and cardiovascular disease has been widely discussed, data on the association between EAT volume and density obtained in an automated manner by computed tomography and the cumulative impact of classical cardiovascular risk factors are much less abundant than those on linear measurement of EAT thickness. In addition, the relative clinical usefulness of EATV and EATD in relation to specific cardiovascular risk factors has not been sufficiently clarified.

The aim of the present study was to assess the significance of computed tomography-derived epicardial adipose tissue volume and density as markers of cardiovascular health and of the effects of cardiovascular disease risk factors in a cohort undergoing CCTA.

## 2. Materials and Methods

### 2.1. Study Design and Study Population

The present study was conducted within the prospective project entitled “Selected quantitative parameters of coronary computed tomography angiography as markers of cardiovascular health”, carried out in the computed tomography laboratory of the 4th Military Hospital in Wroclaw as part of the Wroclaw Medical University project entitled “The importance of selected methods of laboratory, imaging and electrophysiological diagnostics in the assessment of cardiovascular health” [26]. The current analysis included 105 participants and focused exclusively on epicardial adipose tissue parameters.

The inclusion criteria were age ≥ 40 years and a clinical indication for CCTA according to the recommendations of the European Society of Cardiology. The exclusion criteria were coronary artery calcium score (CACS) > 1500, insufficient CCTA image quality, previous coronary intervention, previous myocardial infarction, previous stroke, heart failure, severe systemic disease precluding reliable evaluation, and other standard contraindications to CCTA.

Group size was determined using a sample size calculator. The selection conditions were as follows: population size 3.0 million, fraction size 0.5, maximum error 10%, and confidence level 95%. The required minimum size of the study group was 96; therefore, the final cohort size of 105 was considered sufficient for the planned analyses.

### 2.2. Clinical Assessment, Anthropometric Data, and Laboratory Evaluation

Clinical assessment included medical history, physical examination, anthropometric measurements, blood pressure measurement, and questionnaire-based assessment of smoking status. Age, sex, height, body mass, and BMI were recorded for all participants. Conventional obesity was defined as BMI ≥ 30 kg/m^2^. Arterial hypertension, dyslipidemia, and type 2 diabetes were identified based on previously established clinical diagnoses and/or current treatment, consistent with the approach used in our previous project-related publication [26].

Blood pressure was measured during routine clinical evaluation. Laboratory analyses included measurements of total cholesterol, triglycerides, and fasting glycemia performed with standard laboratory methods. The mean age of the study group was 62.12 ± 11.20 years; 58.1% of participants were women, and 41.9% were men. Mean height was 169.00 ± 9.06 cm, mean body mass was 79.33 ± 15.36 kg, and mean BMI was 27.70 ± 4.52 kg/m^2^. Mean systolic blood pressure was 132.37 ± 16.20 mmHg, mean diastolic blood pressure was 79.07 ± 9.74 mmHg, mean total cholesterol was 191.32 ± 42.94 mg/dL, mean triglycerides were 112.19 ± 38.46 mg/dL, and mean fasting glycemia was 101.91 ± 16.21 mg/dL.

### 2.3. Cardiovascular Risk Factors and Total CVRF Count

The following major cardiovascular risk factors were considered in the present study: older age (>60 years), male sex, BMI-based excess body weight, arterial hypertension, dyslipidemia, type 2 diabetes, and smoking. For the subgroup comparisons the BMI-based category reflected overweight/obesity (BMI ≥ 25 kg/m^2^), which corresponds to the classification used in the source comparative dataset. In contrast, the ROC analyses for obesity were based on the conventional definition of obesity as BMI ≥ 30 kg/m^2^.

In addition to single-factor subgroup analyses, the total number of cardiovascular risk factors (CVRFs) was calculated for each participant. The mean number of CVRFs in the whole group was 3.17 ± 1.42. For comparative analyses, participants were stratified into three subgroups according to total CVRF count: 0–2, 3–4, and 5–7 CVRFs.

### 2.4. Coronary Computed Tomography Angiography Protocol

Cardiac computed tomography was performed using the standard coronary computed tomography angiography protocol with a dual-source 384-slice CT scanner (SOMATOM Force, Siemens Healthcare, Erlangen, Germany). The images were assessed by a certified radiologist with the EACVI Cardiac Computed Tomography Exam and over 10 years of clinical experience.

The coronary artery calcium score was assessed on native-phase CCTA images. Coronary artery disease severity was classified according to the Coronary Artery Disease Reporting and Data System (CAD-RADS), where 0 denotes the absence of coronary artery disease, 1 minimal non-obstructive disease, 2 mild non-obstructive disease, 3 moderate stenosis, 4 severe stenosis, and 5 total coronary artery occlusion.

The CCTA parameters in the study group were as follows: CACS 107.11 ± 240.00, CAD-RADS 0 in 45.7%, CAD-RADS 1 in 20.0%, CAD-RADS 2 in 19.0%, CAD-RADS 3 in 9.5%, CAD-RADS 4 in 3.8%, and CAD-RADS 5 in 1.9%. Mean left ventricular ejection fraction (LVEF) was 69.24 ± 7.01%, mean left ventricular mass (LVM) was 114.58 ± 29.47 g, mean left ventricular end-diastolic volume (LV EDV) was 146.78 ± 46.83 mL, mean left ventricular end-systolic volume (LV ESV) was 45.80 ± 18.94 mL, and mean left ventricular stroke volume (LV SV) was 101.25 ± 32.14 mL.

### 2.5. Epicardial Adipose Tissue Assessment

Epicardial adipose tissue was defined as adipose tissue located between the myocardium and the visceral pericardium. EAT assessment was performed using semi-automatic segmentation on a dedicated post-processing software/workstation: syngo.via Frontier Cardiac Risk Assessment (Siemens Healthcare, Erlangen, Germany). The attenuation range used for EAT identification was −190 to −30 HU. The segmentation was visually verified, and manual correction was performed when necessary. Manual correction was performed based on the consensus of two radiology and imaging specialists experienced in cardiovascular radiology, one of whom is a certified cardiovascular radiologist (EBCR Diploma, EACVI CCT exam, and EACVI CMR exam). The reproducibility of EAT parameter assessment, in the case of manual correction of the automatic assessment based on the consensus of two radiologists, has not been formally assessed. Two epicardial adipose tissue parameters were analyzed: epicardial adipose tissue volume (EATV), expressed in mL, and epicardial adipose tissue density (EATD), expressed in HU. In the whole study group, mean EATV was 284.40 ± 139.62 mL, and mean EATD was −65.46 ± 8.13 HU.

### 2.6. Statistical Analysis

Statistical analysis was performed using Dell Statistica 13.1 software (Dell Inc., Round Rock, TX, USA). Quantitative data are presented as mean ± standard deviation. Depending on the normality of distribution assessed with the Shapiro–Wilk test, parametric or non-parametric methods were used. Comparative analysis of two groups was performed using the *t*-test or the Mann–Whitney U test, as appropriate. In the comparative analysis of more than 2 subgroups, in the case of variables with a normal distribution, ANOVA (one-way parametric) was used, and for variables with a non-normal distribution, the Kruskal–Wallis ANOVA test was used. Qualitative variables are presented as percentages. Correlation and regression analysis were performed to determine relationships between EAT parameters and other variables. Regression analysis was performed using a backward stepwise multivariable approach. Model parameters obtained from the regression analysis were estimated using the least squares method. ROC curve analysis was used to identify cut-off values and evaluate the sensitivity and specificity of EATV and EATD as markers of selected modifiable cardiovascular risk factors. A *p*-value < 0.05 was considered statistically significant.

## 3. Results

The cohort consisted of 105 participants with a mean age of 62.12 ± 11.20 years. Basic clinical parameters in the study group are presented in Table 1.

The mean coronary artery calcium score was 107.11 ± 240.00. According to CAD-RADS, 45.7% of participants had no coronary artery disease, whereas 54.3% had some degree of coronary atherosclerotic involvement. Moderate or more advanced stenosis (CAD-RADS 3–5) was present in 15.2% of participants. Mean EATV was 284.40 ± 139.62 mL, and mean EATD was −65.46 ± 8.13 HU. The results of CCTA are presented in Table 2.

Men had significantly higher EATV than women (344.91 ± 171.34 vs. 240.76 ± 90.16 mL, *p* = 0.01). Participants with overweight/obesity (BMI ≥ 25 kg/m^2^) had significantly higher EATV and significantly lower EATD than participants with BMI < 25 kg/m^2^ (316.56 ± 140.85 vs. 186.69 ± 77.49 mL, *p* = 0.01; −66.72 ± 8.12 vs. −61.62 ± 6.99 HU, *p* = 0.01). Participants with dyslipidemia had significantly higher EATV and significantly lower EATD than participants without dyslipidemia (313.71 ± 119.21 vs. 260.65 ± 151.06 mL, *p* = 0.04; −67.49 ± 7.30 vs. −63.81 ± 8.45 HU, *p* = 0.02). Participants with type 2 diabetes had significantly higher EATV and lower EATD than those without type 2 diabetes (404.85 ± 155.21 vs. 268.86 ± 130.46 mL, *p* = 0.01; −71.83 ± 6.83 vs. −64.63 ± 7.95 HU, *p* = 0.01).

The number of cardiovascular risk factors was significantly associated with both EATV and EATD. EATV increased stepwise from 205.40 ± 134.70 mL in participants with 0–2 CVRFs to 295.45 ± 103.90 mL in those with 3–4 CVRFs and to 396.72 ± 167.75 mL in those with 5–7 CVRFs. The differences were significant for all pairwise comparisons (0–2 vs. 3–4, *p* = 0.02; 0–2 vs. 5–7, *p* = 0.01; 3–4 vs. 5–7, *p* = 0.01). In contrast, EATD showed a stepwise decrease across the same categories, from −61.72 ± 8.18 HU to −66.16 ± 7.48 HU and −70.18 ± 7.38 HU, respectively. These differences were significant for the comparisons of 0–2 vs. 3–4 CVRFs (*p* = 0.03) and 0–2 vs. 5–7 CVRFs (*p* = 0.01), whereas the difference between 3–4 and 5–7 CVRFs did not reach statistical significance (*p* = 0.06). The subgroup analysis is presented in Table 3.

The correlations between EAT parameters and selected quantitative variables are summarized in Table 4.

EATV showed significant positive correlations with age (r = 0.20, *p* = 0.04), BMI (r = 0.54, *p* = 0.01), fasting glycemia (r = 0.46, *p* = 0.01), and total number of CVRFs (r = 0.51, *p* = 0.01). EATD showed significant negative correlations with age (r = −0.26, *p* = 0.01), BMI (r = −0.40, *p* = 0.01), triglycerides (r = −0.44, *p* = 0.02), fasting glycemia (r = −0.49, *p* = 0.01), and total number of CVRFs (r = −0.40, *p* = 0.01).

**Figure 1:**
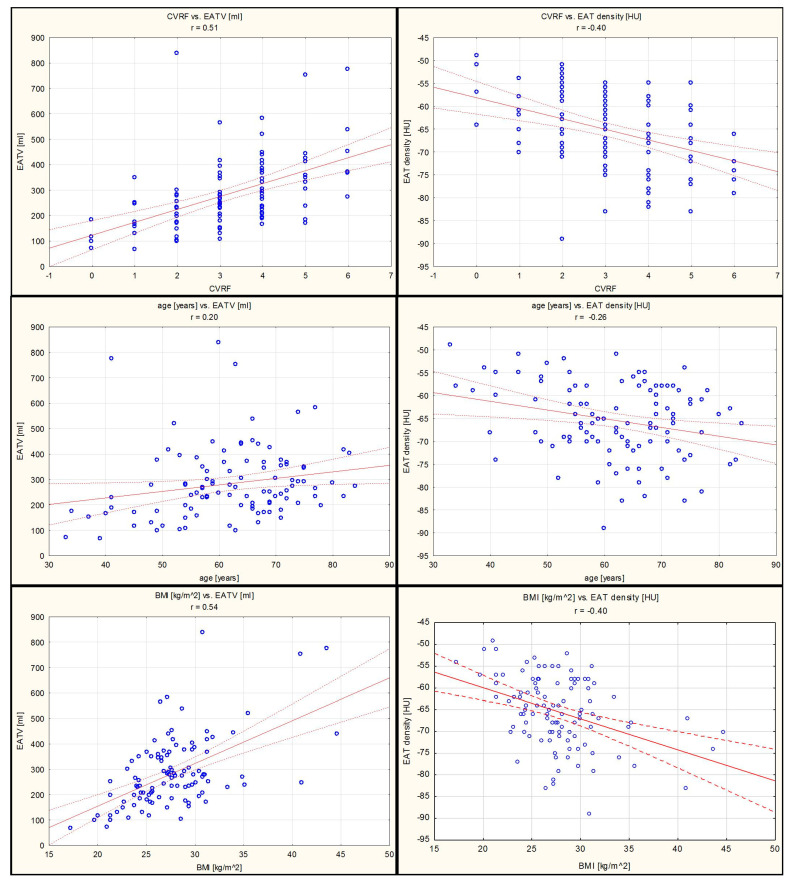

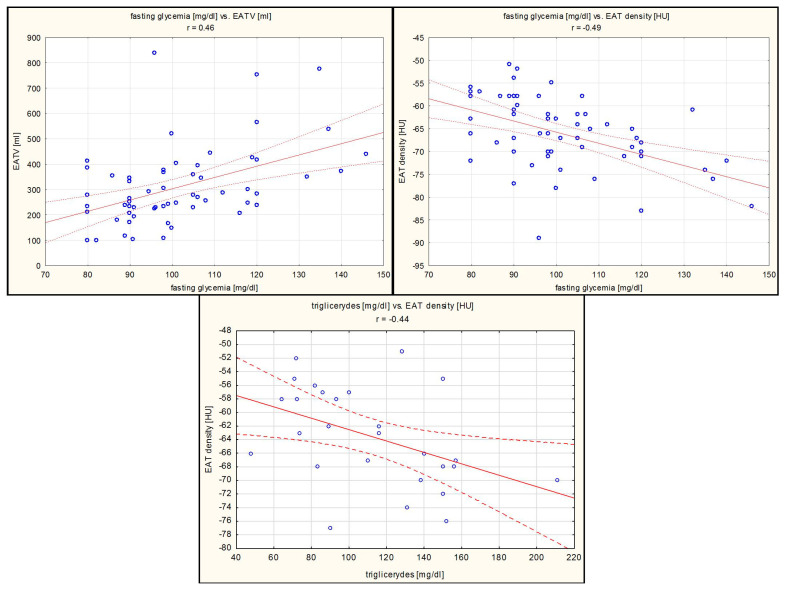
Statistically significant linear relationships in the study group. The solid red line represents the trendline. The dashed red lines indicate the ± 95% confidence interval. The blue circle indicates the data point marker.

The statistically significant correlations between EAT parameters and selected quantitative variables are illustrated in Figure 1.

The results of the multiple regression analysis are presented in Table 5.

A backward stepwise analysis method was used. Cardiovascular risk factors (age, gender, BMI, systolic blood pressure, diastolic blood pressure, total cholesterol concentration, triglyceride concentration, fasting glucose concentration, and smoking) and cardiovascular disease (hypertension, dyslipidemia, and type 2 diabetes) were considered. Statistically significant models of relationships were obtained for factors independently affecting EATV and EATD. Older age, higher BMI, male gender, and dyslipidemia were independent risk factors for higher EATV, while dyslipidemia and type 2 diabetes were independent risk factors for lower EATD.

ROC analysis was performed to compare the predictive value of EATV and EATD variables in the context of various risk factors for cardiovascular disease. ROC analysis identified optimal cut-off values of EATV and EATD for the prediction of selected modifiable cardiovascular risk factors. For EATV, the optimal cut-off values were ≥266.04 mL for obesity, ≥254.05 mL for arterial hypertension, ≥233.69 mL for dyslipidemia, ≥332.68 mL for type 2 diabetes, and ≥450.69 mL for smoking. Among these, the highest Youden index values were observed for obesity (0.505) and type 2 diabetes (0.503), indicating that EATV was most useful as a marker of these two conditions. Specifically, EATV ≥ 266.04 mL identified obesity with a sensitivity of 88.5%, specificity of 62.0%, and accuracy of 68.6%. EATV ≥ 332.68 mL identified type 2 diabetes with a sensitivity of 75.3%, specificity of 75.0%, and accuracy of 75.2%. For EATD, the optimal cut-off values were ≤−67.00 HU for obesity, ≤−55.00 HU for arterial hypertension, ≤−66.00 HU for dyslipidemia, and ≤−72.00 HU for both type 2 diabetes and smoking. The highest Youden index values were noted for type 2 diabetes (0.516) and dyslipidemia (0.319), indicating that EATD was most useful as a marker of these two conditions. Specifically, EATD ≤ −66.00 HU identified dyslipidemia with a sensitivity of 63.8%, specificity of 68.1%, and accuracy of 65.7%. EATD ≤ −72.00 HU identified type 2 diabetes with a sensitivity of 84.9%, specificity of 66.7%, and accuracy of 82.9% (Table 6 and Table 7).

## 4. Discussion

The present study demonstrated that computed tomography-derived epicardial adipose tissue volume and density reflect the effects of cardiovascular disease risk factors on cardiovascular health. The principal findings may be summarized as follows. First, higher EATV and lower EATD were associated with an unfavorable cardiovascular health profile. Second, among the individual risk factors analyzed, the clearest relationships were observed for older age, male sex, overweight/obesity, dyslipidemia, and type 2 diabetes. Third, an increasing total number of major cardiovascular risk factors was accompanied by progressively higher EATV and progressively lower EATD. Fourth, EATV appeared to be the more useful marker in the context of age, male gender, BMI, and type 2 diabetes, whereas EATD appeared particularly informative for dyslipidemia and type 2 diabetes. These findings support the concept that EAT is not only an anatomical fat depot but also a clinically meaningful marker of cardiovascular health. The present results are consistent with the growing body of evidence indicating that both the amount and the qualitative characteristics of epicardial fat are linked to cardiovascular risk and cardiometabolic disease [6,10,11,12].

In the present study, older age was associated with higher EATV and lower EATD in correlation analysis, although between-group differences for the age >60 years subgroup did not reach statistical significance. Older age was an independent risk factor for higher EATV. This pattern is biologically plausible and agrees with previous reports indicating that epicardial fat tends to increase with aging, likely as part of broader age-related changes in visceral adiposity, inflammatory signaling, and metabolic dysregulation [6,24,25]. Aging is accompanied by chronic low-grade inflammation, impaired insulin sensitivity, and progressive remodeling of adipose depots, which may contribute not only to greater EAT accumulation but also to altered tissue composition. The relatively modest strength of the correlations observed in our study suggests that age probably acts in concert with other cardiometabolic factors rather than as an isolated determinant of EAT characteristics.

Men in our cohort had significantly higher EATV than women, while the difference in EATD was not significant. Male gender was an independent risk factor for higher EATV. This finding is in line with the known sex-related differences in visceral adipose tissue distribution and with previous CT- and MRI-based reports showing a tendency toward larger epicardial fat depots in men [5,6,24]. From a pathophysiological perspective, this observation may reflect sex differences in body fat distribution, hormonal milieu, and cardiometabolic phenotype [26]. At the same time, the lack of a significant difference in EATD suggests that the qualitative or compositional properties of EAT may be less strongly sex-dependent than total volume, at least in cohorts of moderate size.

BMI-based excess body weight showed one of the strongest relationships with epicardial adipose tissue in the present study. Participants with overweight/obesity (BMI ≥ 25 kg/m^2^) had both significantly higher EATV and significantly lower EATD. In addition, BMI was the variable most strongly correlated with EATV and also showed a clear inverse correlation with EATD. Higher BMI was an independent risk factor for higher EATV. These findings are consistent with the concept that EAT behaves as a component of visceral adiposity and expands in parallel with general and abdominal fat accumulation [5,6,24,27]. Previous studies have demonstrated that larger EAT depots are associated with adverse metabolic profiles and increased cardiovascular risk, while CT-based volumetric assessment appears superior to isolated thickness measurement in reflecting the overall amount of epicardial fat [20,21,28]. The observed reduction in EATD in participants with excess body weight may indicate qualitative remodeling of epicardial fat, potentially related to increased lipid content, adipocyte hypertrophy, and low-grade inflammation [29]. At the same time, our ROC analysis, which used the conventional definition of obesity as BMI ≥ 30 kg/m^2^, suggests that EATV may be particularly useful in identifying patients with established obesity. This distinction should be kept in mind when interpreting the BMI-related findings of the present study.

Participants with arterial hypertension had numerically higher EATV and lower EATD, but these differences were not statistically significant. This suggests that although hypertension may be accompanied by less favorable EAT characteristics, its isolated association may be weaker than that of excess body weight, dyslipidemia, or diabetes in this cohort. Previous literature has generally supported a link between EAT and hypertension, although the magnitude of this association varies across populations and imaging methods [6,10]. It is possible that, in our cohort, the effect of hypertension was partially mediated by coexisting obesity and metabolic dysfunction, which were highly prevalent [30].

Dyslipidemia was associated with both significantly higher EATV and significantly lower EATD, and triglyceride concentration showed a significant inverse correlation with EATD. Dyslipidemia was an independent risk factor for higher EATV and for lower EATD. These observations are clinically meaningful because they suggest that both the extent and the composition of EAT are linked to atherogenic lipid disturbances. Earlier studies have shown that greater epicardial fat volume is associated with cardiovascular risk factors, including dyslipidemia, and with a more advanced coronary atherosclerotic phenotype [6,10,11,12,31]. In our study, EATD appeared particularly useful for dyslipidemia, as reflected by the highest non-diabetes-related Youden index in the ROC analysis. This may indicate that attenuation-based assessment captures qualitative metabolic alterations of EAT that are closely related to disturbed lipid metabolism.

Type 2 diabetes was the risk factor most strongly associated with both EAT parameters. Participants with diabetes had markedly higher EATV and markedly lower EATD, while fasting glycemia showed significant correlations with both measurements. Type 2 diabetes was an independent risk factor for lower EATD. Moreover, ROC analysis identified type 2 diabetes as one of the conditions best predicted by EATV and the single condition best predicted by EATD. This finding is consistent with previous evidence linking epicardial fat to insulin resistance, glucose metabolism abnormalities, and diabetes-related cardiovascular risk [6,10,32]. Epicardial fat may participate in this relationship through inflammatory and endocrine activity, lipotoxic effects, and local paracrine interactions with the myocardium and coronary vessels [22,23]. Our results suggest that in patients with impaired glucose metabolism, both the amount and the quality of EAT should be considered clinically relevant.

Although smokers in our cohort showed numerically higher EATV and lower EATD, these differences were not statistically significant. Smoking remains a major cardiovascular risk factor, but its relationship with EAT may be more complex and possibly weaker than the relationship observed for excess body weight, dyslipidemia, or diabetes. The low prevalence of smokers in our cohort may also have limited statistical power. Therefore, the absence of significance in the present study should be interpreted cautiously.

One of the most important findings of the present study is the clear association between the total number of cardiovascular risk factors and both EATV and EATD. Participants with a greater number of major risk factors had progressively larger volumes of epicardial fat and progressively lower epicardial fat density. This relationship was supported by both between-group comparisons and correlation analysis. These results suggest that EAT may reflect the cumulative cardiometabolic effect of multiple coexisting risk factors rather than only isolated abnormalities. In practical terms, EAT measurement may therefore provide an integrated imaging-based marker of cardiovascular health. This observation is also conceptually consistent with our previous project-related publication on the left atrioventricular coupling index, in which CT-derived structural parameters reflected the cardiovascular risk profile in a related research setting [33]. In the present analysis, however, the focus was specifically on epicardial adipose tissue, which offers a distinct metabolic and pathophysiological perspective.

Furthermore, previous studies have shown that both increased epicardial fat volume and increased epicardial fat thickness are independent risk factors for coronary artery disease, atrial fibrillation, heart failure, and cardiovascular events, even after accounting for traditional risk scores. This suggests that epicardial fat may serve as a biomarker of residual cardiovascular risk. This indicates the incremental value of EAT parameters in predicting cardiovascular pathology beyond traditional cardiovascular risk factors [14,34,35,36].

The present findings may have some clinical implications. First, EAT assessment can be obtained from routine cardiac CT datasets without the need for additional scanning. Therefore, EAT assessment does not require modification of the standard CCTA protocol and does not involve higher doses of ionizing radiation or a larger volume of administered iodinated contrast. Second, EATV and EATD may provide complementary information: volume appears to reflect the overall amount of epicardial fat, whereas density may reflect tissue composition and metabolic remodeling. Third, the observed associations with higher BMI, dyslipidemia, diabetes, and higher total CVRF may indicate the potential usefulness of EAT assessment in study protocols focused on cardiovascular disease prevention and risk stratification. Although the current data do not justify using EAT parameters as stand-alone diagnostic tools, they may point to their role as informative imaging biomarkers of cardiovascular health.

The study has several limitations. First, this was a single-center, cross-sectional study with a relatively small sample size, which limits generalizability and precludes causal inference and clinical interpretation. It should be noted that the study population was derived from patients undergoing CCTA, which may introduce selection bias. Second, although the study was derived from the same prospective project as our previous publication, the present analysis concerned a modified cohort with partial overlap and addressed a different imaging biomarker within a separate analytical framework. Third, some subgroup sizes, particularly for type 2 diabetes and smoking, were relatively small. Fourth, EAT was evaluated using CT-based attenuation and volumetric analysis, which is robust and reproducible, but CT does not provide the full tissue characterization achievable with dedicated metabolic or histopathological studies. Fifth, ROC analysis was based on cut-off identification and diagnostic performance measures available within the study dataset; external validation in larger cohorts is needed. Sixth, in the study, no discrimination/reclassification analyses were performed, no comparison with established risk models was provided, and no outcome-based analyses were performed. Therefore, statements suggesting incremental prognostic utility should remain cautious and framed as hypothesis-generating rather than demonstrated conclusions. Finally, it should be clearly emphasized that ROC analyses are exploratory in nature and should not be interpreted as clinically validated diagnostic thresholds.

## 5. Conclusions

1. Higher computed tomography-derived epicardial adipose tissue volume and lower computed tomography-derived epicardial adipose tissue density may indicate poorer cardiovascular health.
2. Cardiovascular risk factors associated with epicardial adipose tissue in the present cohort included older age, male sex, higher BMI, dyslipidemia, and type 2 diabetes.
3. A greater total number of cardiovascular risk factors was associated with higher EATV and lower EATD.

## Figures and Tables

**Table 1:** Basic clinical parameters in the study group (*n* = 105).

Parameter	Value
Age, years	62.12 ± 11.20
Male sex, %	41.9
Female sex, %	58.1
Height, cm	169.00 ± 9.06
Body mass, kg	79.33 ± 15.36
BMI, kg/m^2^	27.70 ± 4.52
Arterial hypertension, %	73.3
Antihypertensive drugs	50.5
Systolic blood pressure, mmHg	132.37 ± 16.20
Diastolic blood pressure, mmHg	79.07 ± 9.74
Dyslipidemia, %	44.8
Lipid-lowering drugs	23.8
Total cholesterol, mg/dL	191.32 ± 42.94
Triglycerides, mg/dL	112.19 ± 38.46
Type 2 diabetes, %	11.4
Hypoglycemic drugs	8.6
Fasting glycemia, mg/dL	101.91 ± 16.21
Smoking, %	11.4
CVRF count	3.17 ± 1.42

Values are percentages or mean ± standard deviation. BMI, body mass index; CVRF, cardiovascular risk factor.

**Table 2:** Coronary computed tomography angiography parameters in the study group (*n* = 105).

Parameter	Value
CACS	107.11 ± 240.00
CAD-RADS 0, %	45.7
CAD-RADS 1, %	20.0
CAD-RADS 2, %	19.0
CAD-RADS 3, %	9.5
CAD-RADS 4, %	3.8
CAD-RADS 5, %	1.9
LVEF, %	69.24 ± 7.01
LVM, g	114.58 ± 29.47
LV EDV, mL	146.78 ± 46.83
LV ESV, mL	45.80 ± 18.94
LV SV, mL	101.25 ± 32.14
EATV, mL	284.40 ± 139.62
EATD, HU	−65.46 ± 8.13

Values are percentages or mean ± standard deviation. CACS, coronary artery calcium score; CAD-RADS, coronary artery disease-reporting and data system; EATD, epicardial adipose tissue density; EATV, epicardial adipose tissue volume; LVEF, left ventricular ejection fraction; LV EDV, left ventricular end-diastolic volume; LV ESV, left ventricular end-systolic volume; LVM, left ventricular mass; LV SV, left ventricular stroke volume.

**Table 3:** Epicardial adipose tissue volume (EATV) and epicardial adipose tissue density (EATD) in subgroups determined by cardiovascular risk factors.

Risk Factor/Category	Group	EATV, mL	EATD, HU
Age > 60 years	Yes	299.91 ± 122.10	−66.65 ± 7.97
	No	262.03 ± 122.10	−63.74 ± 8.15
	*p*	0.17	0.07
Male sex	Yes	344.91 ± 171.34	−67.11 ± 9.16
	No	240.76 ± 90.16	−64.26 ± 7.14
	*p*	0.01	0.07
Overweight/obesity (BMI ≥ 25 kg/m^2^)	Yes	316.56 ± 140.85	−66.72 ± 8.12
	No	186.69 ± 77.49	−61.62 ± 6.99
	*p*	0.01	0.01
Arterial hypertension	Yes	296.78 ± 129.29	−65.83 ± 7.28
	No	250.36 ± 162.49	−64.43 ± 10.20
	*p*	0.13	0.44
Dyslipidemia	Yes	313.71 ± 119.21	−67.49 ± 7.30
	No	260.65 ± 151.06	−63.81 ± 8.45
	*p*	0.04	0.02
Type 2 diabetes	Yes	404.85 ± 155.21	−71.83 ± 6.83
	No	268.86 ± 130.46	−64.63 ± 7.95
	*p*	0.01	0.01
Smoking	Yes	339.62 ± 224.88	−68.25 ± 8.62
	No	277.28 ± 124.67	−65.10 ± 8.04
	*p*	0.15	0.21
CVRF count	0–2	205.40 ± 134.70	−61.72 ± 8.18
	3–4	295.45 ± 103.90	−66.16 ± 7.48
	5–7	396.72 ± 167.75	−70.18 ± 7.38
	*p*	0–2 vs. 3–4: 0.02	0–2 vs. 3–4: 0.03
		0–2 vs. 5–7: 0.01	0–2 vs. 5–7: 0.01
		3–4 vs. 5–7: 0.01	3–4 vs. 5–7: 0.06

Values are mean ± standard deviation unless otherwise indicated. The BMI-based subgroup represents overweight/obesity (BMI ≥ 25 kg/m^2^). CVRF, cardiovascular risk factor.

**Table 4:** Correlations between epicardial adipose tissue and other quantitative variables in the study group (*n* = 105).

Variable	EATV, r/*p*	EATD, r/*p*
Age, years	0.20/0.04	−0.26/0.01
BMI, kg/m^2^	0.54/0.01	−0.40/0.01
Systolic blood pressure, mmHg	0.19/0.06	−0.12/0.20
Diastolic blood pressure, mmHg	0.15/0.13	−0.08/0.43
Total cholesterol, mg/dL	0.02/0.89	−0.16/0.26
Triglycerides, mg/dL	0.20/0.31	−0.44/0.02
Fasting glycemia, mg/dL	0.46/0.01	−0.49/0.01
CVRF count	0.51/0.01	−0.40/0.01

Values represent correlation coefficients (r) and *p*-values. BMI, body mass index; CVRF, cardiovascular risk factor.

**Table 5:** Results of estimation for the model obtained in progressive stepwise multivariable analysis of regression.

**Model for: EATV, mL**				
	Regression Coefficient	SEM of Rc	*p*	*p* of the Model
Intercept	−403.064	181.490	0.039	0.002
Age, years	5.334	2.279	0.031	
BMI, kg/m^2^	8.952	3.385	0.016	
Male sex ^a^	89.140	34.447	0.019	
Dyslipidemia ^a^	64.163	30.733	0.041	
**Model for: EATD, HU**				
	Regression Coefficient	SEM of Rc	*p*	*p* of the Model
Intercept	−58.636	1.675	0.001	0.007
Dyslipidemia ^a^	−7.919	2.497	0.004	
Type 2 diabetes ^a^	−7.111	3.074	0.049	

^a^, Dichotomous variable, where 1—yes, 0—no; BMI, body mass index; EATD, epicardial adipose tissue density; EATV, epicardial adipose tissue volume; SEM of Rc, standard error of the mean of regression coefficient.

**Table 6:** Diagnostic performance of epicardial adipose tissue volume as a predictor of selected cardiovascular risk factors.

Metric	Obesity	Arterial Hypertension	Dyslipidemia	Type 2 Diabetes	Smoking
EATV cut-off, mL	≥266.04	≥254.05	≥233.69	≥332.68	≥450.69
Sensitivity	0.885	0.679	0.534	0.753	0.946
Specificity	0.620	0.584	0.745	0.750	0.250
Accuracy	0.686	0.610	0.629	0.752	0.867
Positive predictive value	0.434	0.373	0.721	0.959	0.907
Negative predictive value	0.942	0.833	0.565	0.281	0.375
Likelihood ratio positive	2.329	1.633	2.093	3.011	1.262
Likelihood ratio negative	0.186	0.550	0.625	0.330	0.215
Youden index	0.505 *	0.263	0.279	0.503 *	0.193

* Highest Youden index values. EATV, epicardial adipose tissue volume.

**Table 7:** Diagnostic performance of epicardial adipose tissue density as a predictor of selected cardiovascular risk factors.

Metric	Obesity	Arterial Hypertension	Dyslipidemia	Type 2 Diabetes	Smoking
EATD cut-off, HU	≤−67.00	≤−55.00	≤−66.00	≤−72.00	≤−72.00
Sensitivity	0.769	0.214	0.638	0.849	0.817
Specificity	0.506	0.987	0.681	0.667	0.417
Accuracy	0.571	0.781	0.657	0.829	0.771
Positive predictive value	0.339	0.857	0.712	0.952	0.916
Negative predictive value	0.870	0.776	0.604	0.364	0.227
Likelihood ratio positive	1.558	16.500	1.999	2.548	1.401
Likelihood ratio negative	0.456	0.796	0.532	0.226	0.439
Youden index	0.276	0.201	0.319 *	0.516 *	0.234

* Highest Youden index values. EATD, epicardial adipose tissue density.

## Data Availability

The datasets generated and analyzed during the current study are not publicly available but are available from the corresponding author upon reasonable request.

## Abbreviations

BMI	body mass index
CACS	coronary artery calcium score
CAD	coronary artery disease
CAD-RADS	Coronary Artery Disease Reporting and Data System
CCTA	coronary computed tomography angiography
CT	computed tomography
CVD	cardiovascular disease
CVRF	cardiovascular risk factor
CVRFs	cardiovascular risk factors
EAT	epicardial adipose tissue
EATD	epicardial adipose tissue density
EATV	epicardial adipose tissue volume
HU	Hounsfield unit
LVEF	left ventricular ejection fraction
LV EDV	left ventricular end-diastolic volume
LV ESV	left ventricular end-systolic volume
LVM	left ventricular mass
LV SV	left ventricular stroke volume
MRI	magnetic resonance imaging
